# Identification of Key Residues That Confer *Rhodobacter sphaeroides* LPS Activity at Horse TLR4/MD-2

**DOI:** 10.1371/journal.pone.0098776

**Published:** 2014-05-30

**Authors:** Katherine L. Irvine, Monique Gangloff, Catherine M. Walsh, David R. Spring, Nicholas J. Gay, Clare E. Bryant

**Affiliations:** 1 Department of Veterinary Medicine, University of Cambridge, Cambridge, United Kingdom; 2 Department of Biochemistry, University of Cambridge, Cambridge, United Kingdom; 3 Department of Chemistry, University of Cambridge, Cambridge, United Kingdom; Ajou University, Republic of Korea

## Abstract

The molecular determinants underpinning how hexaacylated lipid A and tetraacylated precursor lipid IVa activate Toll-like receptor 4 (TLR4) are well understood, but how activation is induced by other lipid A species is less clear. Species specificity studies have clarified how TLR4/MD-2 recognises different lipid A structures, for example tetraacylated lipid IVa requires direct electrostatic interactions for agonism. In this study, we examine how pentaacylated lipopolysaccharide from *Rhodobacter sphaeroides* (RSLPS) antagonises human TLR4/MD-2 and activates the horse receptor complex using a computational approach and cross-species mutagenesis. At a functional level, we show that RSLPS is a partial agonist at horse TLR4/MD-2 with greater efficacy than lipid IVa. These data suggest the importance of the additional acyl chain in RSLPS signalling. Based on docking analysis, we propose a model for positioning of the RSLPS lipid A moiety (RSLA) within the MD-2 cavity at the TLR4 dimer interface, which allows activity at the horse receptor complex. As for lipid IVa, RSLPS agonism requires species-specific contacts with MD-2 and TLR4, but the R2 chain of RSLA protrudes from the MD-2 pocket to contact the TLR4 dimer in the vicinity of proline 442. Our model explains why RSLPS is only partially dependent on horse TLR4 residue R385, unlike lipid IVa. Mutagenesis of proline 442 into a serine residue, as found in human TLR4, uncovers the importance of this site in RSLPS signalling; horse TLR4 R385G/P442S double mutation completely abolishes RSLPS activity without its counterpart, human TLR4 G384R/S441P, being able to restore it. Our data highlight the importance of subtle changes in ligand positioning, and suggest that TLR4 and MD-2 residues that may not participate directly in ligand binding can determine the signalling outcome of a given ligand. This indicates a cooperative binding mechanism within the receptor complex, which is becoming increasingly important in TLR signalling.

## Introduction

The Toll-like receptor (TLR) family is the most widely studied pattern recognition receptor family, comprising 13 mammalian members, of which 10 are expressed in humans [1]. Toll-like receptor 4 (TLR4) is expressed on the plasma membrane and endosomes to sense lipopolysaccharide (LPS) from Gram-negative bacteria, requiring the co-receptor MD-2 to bind the lipid component of LPS (lipid A) and form the active complex [2], [3]. The crystal structure of human and mouse TLR4/MD-2 bound to *E. coli* LPS (ECLPS) demonstrates a heterotetrameric structure, comprising two TLR4, two MD-2 and two LPS molecules [4], [5]. Five of the six lipid A acyl chains sit deep within the MD-2 pocket, and the sixth (R2 chain) protrudes from the MD-2 pocket to form part of the dimerisation interface with the opposing TLR4 (denoted thereafter TLR4*).

**Figure 1:**
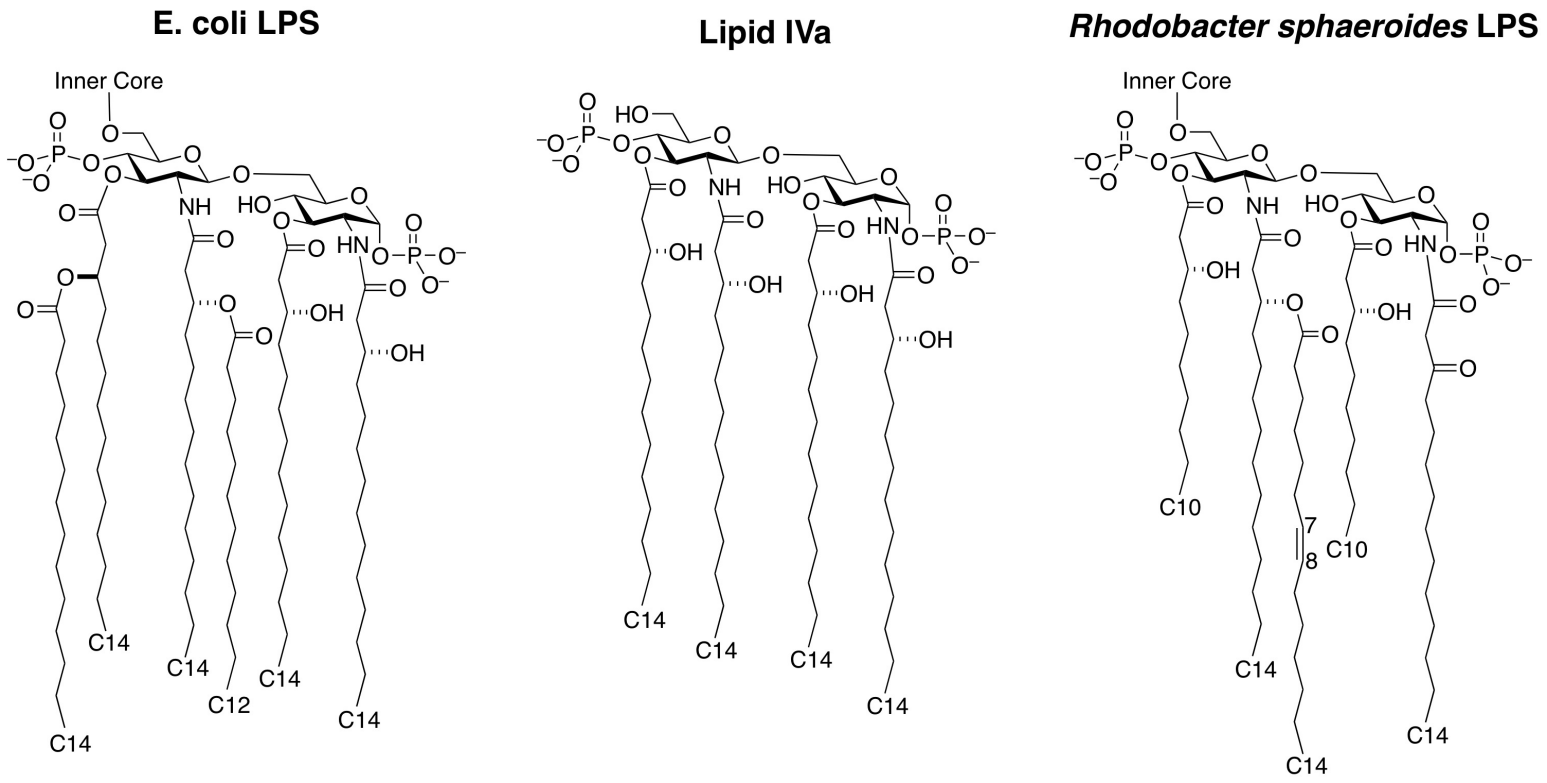
Chemical structures of lipid A derivatives. A) Lipid A from *E. coli*. B) Lipid A synthesis intermediate lipid IVa. C) Lipid A from *Rhodobacter sphaeroides*.

TLR4/MD-2 from all domestic mammalian species recognises hexaacylated lipid A from *E. coli* (figure 1A) as an agonist, but structural variability in lipid A from other Gram-negative organisms alters their efficacy (maximum stimulation, E_max_) and potency (half maximum effective concentration, EC50) at the receptor complex. A reduction in acyl chain number from six to four in the lipid A synthesis intermediate lipid IVa (figure 1B) makes this compound an antagonist at TLR4/MD-2, but only in humans. At horse and mouse TLR4/MD-2, lipid IVa is an agonist, with residues R385 in horse TLR4, and K367 and R434 in mouse TLR4, being important for agonist activity [6], [7], [8], [9]. Eritoran, a tetraacyl chain lipid A analogue derived from the structure of *Rhodobacter sphaeroides* LPS (RSLPS), is an antagonist at human, mouse and horse TLR4/MD-2.

Antagonist-conformation crystal structures of human TLR4/MD-2 proteins bound to lipid IVa and eritoran demonstrate gross similarities to each other and significant differences to lipid A [10], [11]. Their relative orientations in the MD-2 lipid-binding pocket are inverted, with either the 4′-phosphate (4′-PO_4_) in the antagonist conformation or the 1-PO_4_ in the agonist conformation facing the LPS-binding motif of MD-2 [4], [5], [10], [11], [12]. Both ligands have only four acyl chains, which are fully accommodated within the MD-2 binding pocket. A recent crystal structure of lipid IVa bound to mouse TLR4/MD-2 demonstrates that lipid IVa can nevertheless form similar interactions to *E. coli* lipid A in the presence of appropriate surface charge distribution on TLR4/MD-2 [5]. This results in a 4–7 Å difference in glucosamine backbone position along a 180° rotation between human- and mouse-bound lipid IVa conformations. The positioning of the ligand triggers conformational switching in MD-2 and dimerisation of TLR4 for agonists, and apparently fails to do so in the case of antagonists. It is not clear, however, whether novel lipid A structures will also share similar interacting properties. Subtle differences exist in the interaction patterns of lipid IVa and lipid A at the TLR4/MD-2 complex, and these probably underlie the small differences in efficacy between the two ligands at mouse TLR4/MD-2 [5]. The mouse lipid IVa-TLR4-MD-2 complex is monomeric in solution, but dimeric within crystals as a result of crystal packing. At the molecular level, the mouse TLR4 dimer interface residues K367* and R434* both contact the lipid IVa and lipid A 1-PO_4_ groups, whereas an extra hydrogen bond forms between lipid A/TLR4. This extra bond is, therefore, most likely required for maximal ligand efficacy.

Lipid A from *Rhodobacter sphaeroides* (RSLA; figure 1C) has five acyl chains, with an extended and unsaturated R2″ acyl chain, two shortened chains (R3 and R3′), and absent R3″ chain in comparison to *E. coli* lipid A [13]. RSLPS is an antagonist at human and mouse TLR4/MD-2, but an agonist at horse TLR4/MD-2. The agonist activity of RSLPS, like lipid IVa, requires both species-specific TLR4 and MD-2 [14]. In this study, we identify the regions of TLR4 and MD-2 required for RSLPS agonist activity. Two specific amino acid residues in horse TLR4 are required for full RSLPS signalling (R385 and P442). RSLPS has increased efficacy compared to lipid IVa, which is most likely due to differences in positioning between RSLA and lipid IVa affecting how the 1-PO_4_, 4′-PO_4_ and R2 chain interact with TLR4 and TLR4*. Interestingly, residues critical in conferring RSLPS activity also appear to regulate basal activity at the horse receptor complex.

## Materials and Methods

### DNA constructs

The plasmid vector pEFIRES was generated by removal of the BamHI restriction site from pEFIRES-P as described previously [15]. Plasmids containing cDNA were as follows: human and horse TLR4 (hTLR4 and eqTLR4 respectively) in pcDNA3; human and horse CD14 (hCD14 and eqCD14 respectively) in pcDNA3; human and horse MD-2 (hMD-2 and eqMD-2 respectively) in pEFIRES. Chimeric receptors with domain exchange between species were generated by overlap extension PCR using Phusion High-Fidelity DNA Polymerase (Finnzymes). Mutant receptors with single point mutations were generated by site-directed mutagenesis using Pfu polymerase (Fermentas).

### Cell culture and transient transfection

Human Embryonic Kidney 293 (HEK293) cells were purchased from ATCC and maintained in Dulbecco's Modified Eagle's medium (Sigma) containing 10% foetal calf serum (FCS; Hyclone), 2 mM l-glutamine, 100 iu/ml penicillin and 100 µg/ml streptomycin (referred to as complete culture medium). Cells were plated onto a 96 well plate at 3×10^4^ cells/well and transfected two days later. Differing combinations of plasmid-cDNA together with the reporter vectors pNF-κB-luc (Clontech), encoding a firefly luciferase under an NF-κB promoter, and phRG-TK (Promega), encoding a constitutively expressed *Renilla* luciferase, were transfected into the cells using jetPEI (Polyplus) according to the manufacturer's instructions. Briefly, plasmids were mixed in the desired amounts before being added to jetPEI in 150 mM NaCl. Mixtures were incubated for 30 minutes before being diluted in complete culture medium. Old medium was removed from plated cells and replaced with the plasmid mixtures. Plasmid-cDNA amounts per well were as follows: TLR4-pcDNA3 1 ng, MD-2-pEFIRES 1 ng, CD14-pcDNA3 1 ng. Reporters were added at 10 ng/well pNF-κB-luc and 5 ng/well phRG-TK. To maintain a constant amount of DNA (100 ng) per well, DNA amounts were adjusted with empty pcDNA3. Cells were stimulated two days after transfection.

### Lipopolysaccharides

Phenol-extracted LPS from *E. coli* O157:B8 (ECLPS) was purchased from Sigma. Ultrapure LPS from *Rhodobacter sphaeroides* (RSLPS) was purchased from Invivogen. Lipid IVa was purchased from Pepta Nova. RSLPS and ECLPS were reconstituted in sterile endotoxin-free water (Sigma) to 1 mg/ml. Lipid IVa was reconstituted in sterile DMSO to 1 mg/ml. All LPS derivatives were sonicated prior to freezing at −20°C in aliquots and then re-sonicated for one minute following thawing before use.

### Stimulation of cells

**Figure 2:**
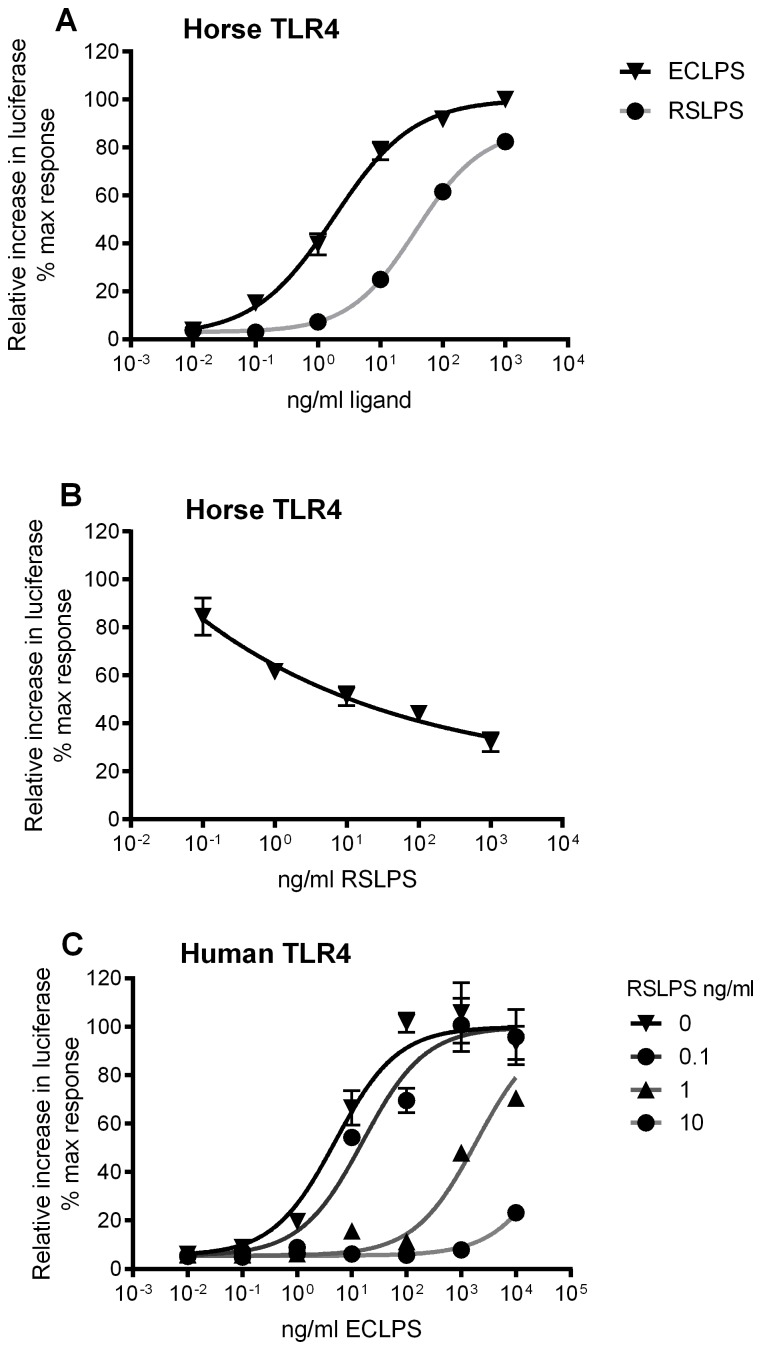
RSLPS is a partial agonist at horse TLR4/MD-2 and competitive antagonist at human TLR4/MD-2. HEK293 cells were transiently transfected with horse or human TLR4, MD-2 and CD14, together with reporter constructs NF-κB-luc and phRG-TK. Cells were stimulated 48 hours later for 6 hours. Data are from a representative experiment (n = 3 experiments) and expressed as triplicate mean ±SEM for that experiment, relative to the maximum ECLPS response. A and B) Horse TLR4/MD-2/CD14-transfected cells were stimulated with increasing concentrations of RSLPS or increasing concentrations of ECLPS (A), or increasing concentrations of RSLPS+10 ng/ml ECLPS (B). C) Human TLR4/MD-2/CD14-transfected cells were stimulated with increasing concentrations of ECLPS in the presence of 0, 1, 10 and 100 ng/ml RSLPS.

ECLPS, RSLPS and lipid IVa were diluted in Dulbecco's Modified Eagle's medium containing 0.1% FCS, 2 mM l-glutamine, 100 iu/ml penicillin and 100 µg/ml streptomycin (referred to as 0.1% FCSM). 0.1% FCS was added to stimulation media to provide a source of LPS binding protein without addition of excessive bovine MD-2 [15]. ECLPS was used at 10 ng/ml, RSLPS was used at 100 ng/ml and lipid IVa was used at 1 µg/ml final concentration; these concentrations were determined by dose-response analysis (figure 2A and 2C; not shown for lipid IVa) to give a maximal agonist response by RSLPS and lipid IVa at horse TLR4 and a strong inhibition of ECLPS at human TLR4. Medium containing transfection reagent was removed from cells and replaced with one of 0.1%FCSM alone, RSLPS, ECLPS, ECLPS+RSLPS, lipid IVa or lipid IVa+ECLPS, and cells incubated for 6 hours at 37°C/5% CO_2_. Media were then removed and cells washed with 200 µl warmed PBS. Diluted Passive Lysis Buffer (PLB; Promega) was added at 50 µl per well and luciferase activity determined using the Dual-Luciferase Assay kit (Promega) according to the manufacturer's instructions.

### Receptor alignments and sequence variation analysis

Receptor sequences for human, horse, mouse, cat and rat TLR4 were entered into the ClustalW multiple sequence alignment tool to generate a Protein Information Resource format alignment. Sequences were then entered into the online TraceSuite ii server (http://mordred.bioc.cam.ac.uk/~jiye/evoltrace/evoltrace.html) to assess sequence variation and define residues present in the horse only.

### Structural templates for human TLR4-MD-2

The crystal structure of human MD-2 in complex with lipid IVa was chosen to represent a typical inactive conformation of antagonist-bound MD-2 [11]. The crystal structure of human TLR4/MD-2 was used to represent the active conformation of TLR4/MD-2 [4], [5]. The coordinates of lipid IVa and LPS were removed from the Protein Data Bank files 2E59 and 3FXI respectively. The monomeric MD-2 structure extracted from 2E59 and the dimeric TLR4/MD-2 structure from 3FXI were then used in the docking experiments.

### Homology modelling of horse TLR4-MD-2

Horse TLR4 and MD-2 proteins were modelled using the human and mouse complexes [4], [5]. Homology models were built using Modeller software [16]. The best model was chosen based on the lowest energies and violations as calculated by Modeller. A further quality assessment of the model was performed using Verify3D and also Ramachandran plot analysis, which was carried out with Molprobity [17], [18].

### *In silico* construction of RSLA

The crystal structures for eritoran, as observed in complex with human MD-2, an N-terminal truncation of TLR4 with ECLPS in complex with human MD-2, and the non-truncated ectodomain of human TLR4 were used as templates to build RSLA molecules [4], [10]. Eritoran and the ECLPS structure were extracted from the corresponding PDB files and processed in Sybyl version 8.1.1 (Tripos), in order to generate RSLA. Sybyl software allowed editing of existing moieties and modelling of additional chemical groups in the first instance. Geometry optimisation was then carried out using the Powell minimisation method, with initial optimisation based on the Simplex method, and with a gradient of 0.05 kcal/mol and a maximum of 100 cycles of iteration. Partial charges were computed based on the Gasteiger-Hückel charge method.

### Molecular docking

Molecular docking of RSLA molecules was carried out using the Autodock 4.0 software package [19]. The receptor models of inactive MD-2 and active TLR4/MD-2 were treated as rigid molecules. Although engagement of two agonist ligands are required to generate the active TLR4/MD-2 heterotetrameric complex, only one docking event was calculated, in keeping with the binding symmetry. The lipid A acyl chains were kept semi-flexible with the maximum allowed torsion angles during docking, ie. 32 torsions. The Autogrid parameters were computed on a grid size 42×66×70 Å^3^, with a spacing of 0.375 Å. The grid was centred on lipid A in the hydrophobic cavity of active MD-2 at x = +29.008; y = −8.418; z = +17.182. Unless stated otherwise, parameters were kept at their default values. Hundred docking poses of the ligand were generated using the Lamarckian version of genetic algorithm searches. The protocol is based on the optimisation of an initial population of 150 randomly-placed individuals, a maximum number of 2.5 million energy evaluations, a maximum number of 27,000 generations with a mutation rate of 0.02, and a cross-over rate of 0.8. The lowest energy complexes were analysed in depth. Structural images were generated in PyMol, and atomic distances measured using the PyMol measurement wizard.

### Statistical methods

Experiments were repeated three times to ensure qualitative repeatability, and data are expressed as a representative (single) experiment. Data except figure 2C are shown as a percentage of the maximum horse TLR4/MD-2 ECLPS response for comparison between different experiments [9], [20], [21]. Figure 2C is expressed as a percentage of the maximum human ECLPS response. Statistical analyses were performed on individual experiments. Error bars are defined as the standard error of the mean (SEM) for the representative experiment, and calculated using triplicate replicates for that value within that experiment. Dose response curves were fitted by non-linear regression to a sigmoidal dose-response (variable slope) model, using GraphPad Prism software, to allow determination of EC50 and the associated 95% confidence intervals (CI). For comparison of curves, best-fit values for maximum stimulation and logEC50 were compared using F tests. Bar graph values were compared using an unpaired two-tailed students't-test with Welch's correction for unequal variance (as required), and with Bonferroni correction for multiple comparisons.

## Results

### Residues 55-107 of MD-2 and LRR14-17 of TLR4 are important for the agonist activity to RSLPS

Dose-response assays confirmed RSLPS activates horse TLR4/MD-2, but also dose-dependently inhibits ECLPS activity (figures 2A and 2B). Maximum stimulation by RSLPS at horse TLR4, as determined by nonlinear regression, was significantly lower than by ECLPS (88% ECLPS activity) (figure 2A). These data show that RSLPS is a partial agonist with high efficacy at horse TLR4. The EC50 for RSLPS at horse TLR4 was 37 ng/ml (95% CI 33–42 ng/ml), which is significantly greater than for ECLPS at 1.8 ng/ml (95% CI 1.4–2.3 ng/ml) (figure 2A). Our work previously defined the EC50 for lipid IVa at horse TLR4 as 5 ng/ml, and lipid IVa efficacy as significantly lower than ECLPS at 55.5% activation [15]. RSLPS would seem to have a lower potency and higher efficacy than lipid IVa. The exact molecular weight is not known for RSLPS, in particular since non-synthetic LPS is heterogeneous, however we estimate it to be a 10-fold order of magnitude (10–20 kDa) compared to lipid IVa (1.4 kDa). The potency of RSLPS is, therefore, within the same range as that of lipid IVa (approximately 1.8–3.7 nM compared to 3.6 nM, respectively). RSLPS dose-dependently inhibited ECLPS activation of human TLR4/MD-2 without inducing any agonist response, confirming it is a competitive TLR4 antagonist in this species (figure 2C) [22].

**Figure 3:**
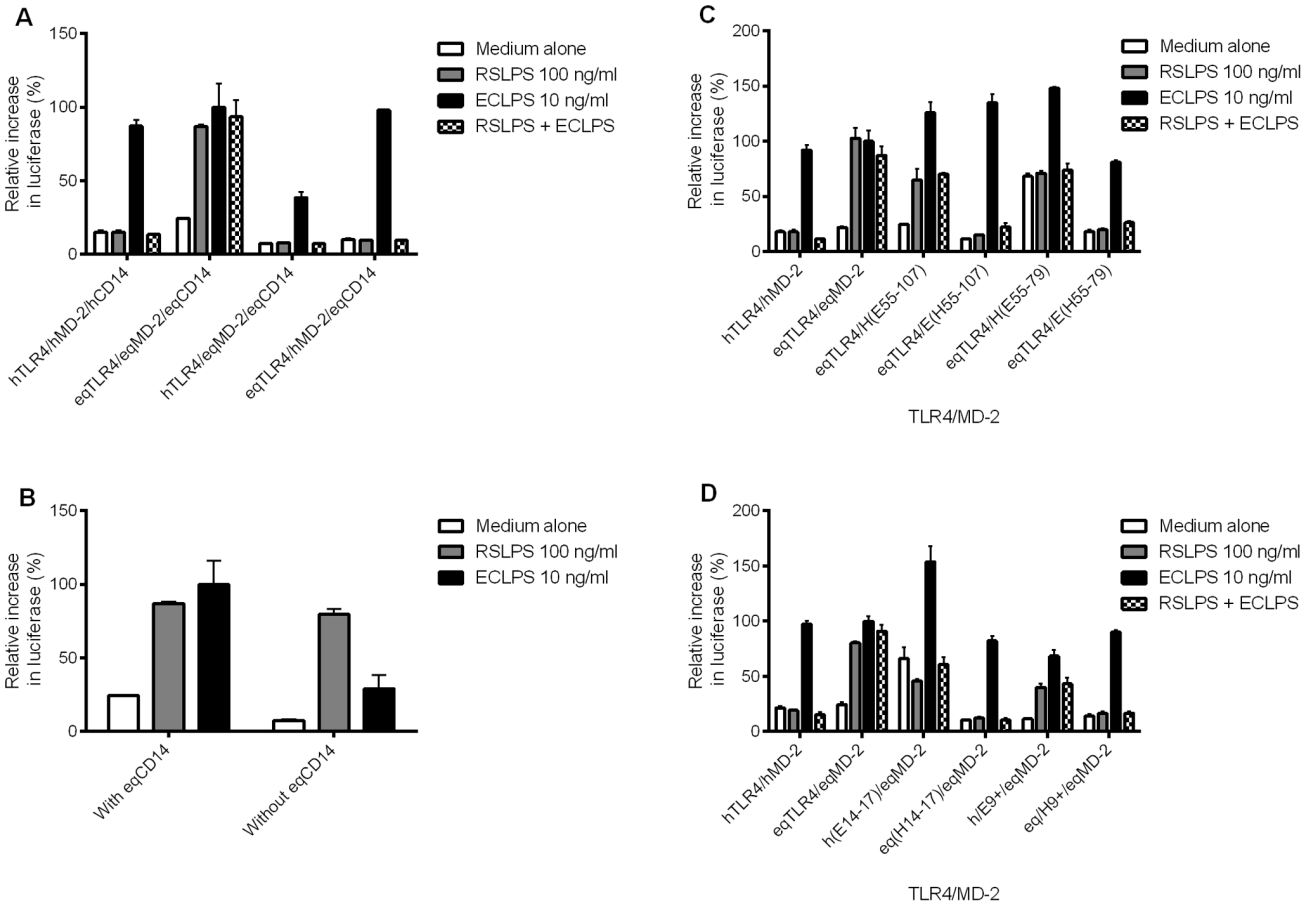
RSLPS requires specific residues within horse MD-2 and TLR4, yet is independent of CD14. HEK293 cells were transiently transfected with combinations of human and horse TLR4 and MD-2, with or without horse CD14, and reporter constructs NF-κB-luc and phRG-TK. Cells were stimulated 48 hours later for 6 hours with 100 ng/ml RSLPS, 10 ng/ml ECLPS, 100 ng/ml RSLPS+10 ng/ml ECLPS, or medium alone. Data are from a representative experiment (n = 3 experiments) and expressed as triplicate mean ±SEM for that experiment, relative to the maximum ECLPS response. A) Cells were transfected with different combinations of human and horse TLR4 and MD-2. B) Horse TLR4/MD-2 was transfected with and without CD14. C) MD-2 mutants were transfected with horse TLR4/CD14. D) TLR4 mutants were transfected with horse MD-2/CD14.

RSLPS stimulation, as shown previously, required both horse TLR4 and horse MD-2 to induce agonist activity (figure 3A) [14]. CD14 sensitised ECLPS activation of TLR4/MD-2, but had no effect on the agonist activity of RSLPS (figure 3B).

Residues 57–107 of horse MD-2 are essential for signalling to lipid IVa [9]. Aligned human and horse MD-2 sequences are shown in figure S1. A chimera in which horse MD-2 residues 55–107 are replaced with the equivalent human MD-2 residues, when transfected with horse TLR4, failed to signal to RSLPS (figure 3C). The complementary chimera containing residues 55–107 of horse MD-2 within the human MD-2 framework signalled to RSLPS, suggesting these residues are as important for signalling to RSLPS as they are for lipid IVa signalling. Neither MD-2 chimera signalled to RSLPS with human TLR4 (not shown). Within 57–107, residues 57–79 of horse MD-2 are essential for lipid IVa signalling, and are sufficient to confer lipid IVa agonist activity onto human MD-2 [15]. A loss of agonist activity was seen with RSLPS, as for lipid IVa, when residues 55–79 of horse MD-2 were replaced with the equivalent human residues (figure 3C). This region of MD-2 was not, however, able to confer RSLPS signalling onto human MD-2. Unlike for lipid IVa, residues within 80–107 are therefore also required for full RSLPS agonist activity. These data suggest that RSLPS may form a wider interface with horse MD-2 than lipid IVa.

LRR14-17 (residues 373–476) of horse TLR4 are critical for lipid IVa agonist activity, and this region of horse TLR4 is sufficient to confer signalling to lipid IVa onto human TLR4 [9]. Sequence alignments for this region are shown in figure S2. Using a chimera of horse TLR4 containing the human LRR14-17 (residues 372–475), co-expressed with horse MD-2, switched RSLPS to an antagonist as observed for lipid IVa (figure 3D). The complementary chimera, however, did not signal to RSLPS when transfected with horse MD-2. Neither construct signalled to RSLPS with human MD-2 (data not shown). These data suggest LRR14-17 are important for RSLPS agonist activity, but residues outside LRR14-17 also contribute to RSLPS signalling. This was confirmed by replacing LRR9 onwards of human TLR4 with the corresponding horse residues, which resulted in conferring responsiveness to RSLPS when transfected with horse MD-2 (figure 3D). The agonist activity of RSLPS at horse TLR4 was lost when LRR9 onwards was replaced with the corresponding human residues and transfected with horse MD-2. Replacing horse TLR4 LRR9-13 or 18–20 with the human residues did not affect signalling to RSLPS, nor did the horse residues confer signalling onto human TLR4 (data not shown). This suggests species-specific amino acids in LRR9-13 and 18–20 contribute to RSLPS signalling only in the presence of horse LRR14-17. None of these chimeras signalled to RSLPS when transfected with human MD-2 (not shown).

### Horse models of TLR4-MD-2 possess unique structural features resulting in distinctive lipid-binding cavities compared to human and mouse structures

The sequences of horse TLR4 and MD-2 proteins are more closely related to human than to mouse, even though the functionality of the complex stands out compared to both. As the crystal structures are known for both human and mouse active complexes we decided to generate models for the horse complex based on both of them independently (and thus generating a human-based model and a mouse-based one) and, together in an averaged model [4], [11]. The root mean square deviations between the different models are within 2 Å (0.6 Å for the averaged model and 1.8 Å for the mouse model compared to the human one). For TLR4, the Cα trace deviates in the convex region leading to a change in the overall curvature of the receptor's ectodomain. The alignment of the central domain (where the dimerisation interface lies) results in shifts of 3–7 Å at the N- and C-terminal ends respectively between human and mouse models (the C-terminal end of the ectodomain being the external juxtamembrane domain of the receptor). In MD-2, loops between the second and the third β-strand L2-3 (residues 37–44), the loop that bares the LPS-binding motif between the seventh and the eight β-strand L7-8 (residues 122–129), and the following one L8-9 (residues 140–143) adopt different conformations, whereas the Cα trace of the secondary structural elements are overall well conserved. These variations in structure lead to a cavity of 1250 Å^2^/2510 Å^3^ for the mouse-based horse model against 1200 Å^2^/2305 Å^3^ for the human-based horse model, and represent a 10% difference in volume. The human MD-2 cavity (1100 Å^2^/2370 Å^3^) is largest of all, with the mouse cavity smallest (1060 Å^2^/2090 Å^3^). Docking was performed on the averaged model (1200 Å^2^/2330 Å^3^).

### Docking studies to TLR4/MD-2 generates biologically relevant ligand poses

We used molecular docking to propose how RSLPS interacts with MD-2 and differs between human and horse TLR4-MD-2 complexes. An initial RSLPS-bound model was generated in which the acyl chains extend away from the phosphorylated diglucosamine core. Docking generated poses that accommodate the longest chain (R2″) by folding it back on itself to fit into the hydrophobic pocket in MD-2, which is also seen for the C18 chain of eritoran [10]. As for ligands previously studied at a structural level, the head group is exposed to the solvent and interacts with charged residues at the opening of the MD-2 cavity.

RSLPS could adopt two orientations regarding positioning of the phosphorylated diglucosamine core, with the 1-PO_4_ group pointing toward either the primary TLR4 binding site or the dimeric interface. The latter is observed for the horse TLR4-MD-2 active complex, whereas the former is observed for human complexes with either MD-2 alone or both TLR4/MD-2. Interestingly, areas of contact made by the lipid moiety of RSLPS also shift within the MD-2 pocket. In human complexes, the lipid tails lean more towards the β-sheet formed by strands 5 and 6. In the horse complex, the acyl chains are found in the vicinity of the opposite β-sheet formed by strands 4–7 and the LPS binding motif mentioned earlier, L7-8, that contains the critical residue F126. Comparisons of human- and horse-bound RSLPS exemplify how subtle changes in ligand binding lead to crucial differences in biological activity.

### Docking highlights species-specific differences in lipid A positioning

**Figure 4:**
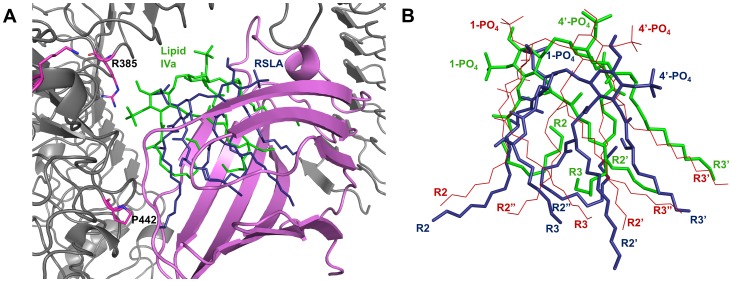
RSLA and lipid IVa sit differently within the MD-2 pocket. A) Docking models of RSLA and lipid IVa bound to horse TLR4/MD-2 were overlaid to assess ligand and receptor positioning. The acyl chains of RSLA (blue) sit more deeply in the MD-2 (pink) pocket than lipid IVa (green), and the R2 chain of RSLA protrudes from the MD-2 pocket to contact TLR4* (grey). The 1-PO_4_ is also moved away from TLR4 due to lowering of the diglucosamine backbone. B) Overlay of RSLA (blue; horse model), lipid IVa (green; horse model) and lipid A (red; human crystal) in situ in the MD-2 pocket. TLR4 and MD-2 have been removed for clarity. The PO_4_ groups and acyl chains of all three ligands sit somewhat differently to one another within the pocket. Lipid A and RSLA appear to occupy a similar volume within the pocket.

Structural models of RSLA bound to inactive MD-2 and active TLR4/MD-2 dimer complex were generated for human and horse complexes and aligned to available crystal structures of ECLPS and lipid IVa complexes with human and mouse proteins (figure 4A). One hundred 3D arrangements were generated per calculation. Only the biologically relevant ones were analysed (ie. lipid tails within the hydrophobic cavity of MD-2). Overall, TLR4 and MD-2 proteins models and crystal structures from different species superimpose well (with a root mean square deviation <2 Å over about 9,600 atoms). Comparison of modelled RSLA to experimentally characterised complexes showed that all ligands have slightly different conformations (figure 4B). RSLA and lipid A acyl chains follow a broadly similar shape, and fill a greater volume of the MD-2 pocket than lipid IVa. It is clear, however, that the acyl chains of both RSLA and lipid IVa fail to follow the acyl chains of lipid A exactly. These differences cannot be explained by the different species of MD-2, since the overlap of lipid A-bound human and mouse MD-2 is greater than that of the two horse MD-2 3D models. Concomitantly with the position of the F126 loop, significant differences are observed in the positioning of the phosphate groups of the ligands. In particular, the 4′-PO_4_ groups are variably positioned facing either the primary TLR4 side or towards the dimeric TLR4* direction. In the horse active complex, the R2 chain of RSLA, like lipid A, protrudes from the MD-2 pocket to contact TLR4*, whereas R2 of lipid IVa is folded back into the pocket and fails to contact the dimerisation interface (figure 4A and 4B). The tip of RSLA R2 points towards P442* of horse TLR4* at a distance suitable for van der Waals contacts (<4.5 Å). The other acyl chains of RSLA penetrate more deeply into the MD-2 pocket, with a concomitant lowering of the diglucosamine backbone and rotation of the 1-PO_4_ away from the primary TLR4 site.

### R385 and P442 of horse TLR4 are required for the agonist activity of RSLA

**Figure 5:**
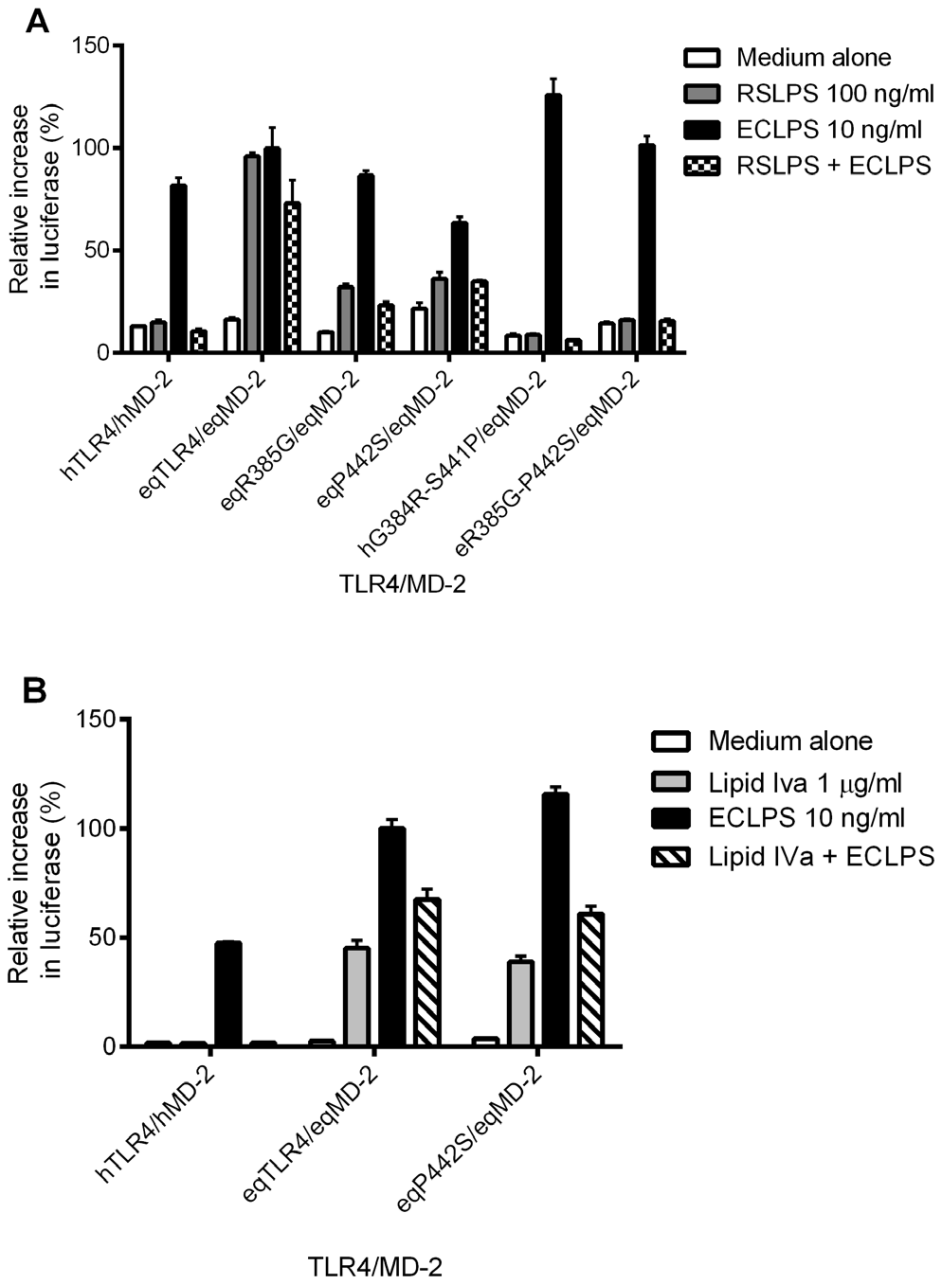
RSLPS activity requires the presence of both R385 and P442 in horse TLR4. HEK293 cells were transiently transfected with combinations of human and horse TLR4 and MD-2, together with horse CD14 and reporter constructs NF-κB-luc and phRG-TK. Cells were stimulated 48 hours later for 6 hours. Data are from a representative experiment (n = 3 experiments) and expressed as triplicate mean ±SEM for that experiment, relative to the maximum ECLPS response. A) TLR4 point mutants were transfected with horse MD-2/CD14 and stimulated with 100 ng/ml RSLPS, 10 ng/ml ECLPS, 100 ng/ml RSLPS+10 ng/ml ECLPS or medium alone. B) TLR4 point mutants were transfected with horse MD-2/CD14 and stimulated with 1 µg/ml lipid IVa, 1 µg/ml lipid IVa+10 ng/ml ECLPS, or medium alone.

Molecular modelling identified that R385 and P442 were likely to be important residues for the agonist activity of RSLPS, and of these R385 of horse TLR4 is essential for lipid IVa agonist activity [9]. We therefore transfected HEK293 cells with human TLR4 G384R (hG384R) or horse TLR4 R385G (eqR385G), together with horse MD-2, and stimulated the cells with RSLPS. A modest reduction in the agonist activity of RSLPS was observed with eqR385G, but the mutation did not abrogate the signal as was seen previously for lipid IVa (figure 5A) [9]. Human TLR4 G384R did not signal to RSLPS with horse MD-2 and remained an antagonist of ECLPS. Neither construct signalled to RSLPS with human MD-2 (not shown). R385 is therefore involved in RSLPS agonist activity, but other residues are also required. Mutation of P442 to the corresponding human residue (P442S) also conferred a modest reduction in RSLPS signal. It did not, however, affect the signal to lipid IVa (figure 5B), confirming that the predicted difference in positioning of the ligand R2 chains and their interactions with the TLR4* interface are important. R385G and P442S single mutations of horse TLR4 each caused a modest reduction in RSLPS signal, yet neither alone caused abrogation. A horse TLR4 mutant was therefore constructed containing both of these mutations. Combined mutation of R385G and P442S caused a complete loss of the RSLPS signal, confirming the importance of these two residues in RSLPS agonist activity at horse TLR4 (figure 5A). The complementary double mutation G384R-S441P within human TLR4 was unable to confer signalling to RSLPS, however, indicating other residues are also involved in recognition of RSLPS as an agonist.

## Discussion

A key question generated by solving the LPS-bound TLR4/MD-2 structures was whether there would be similar molecular determinants when other lipid A variants bound to the receptor complex [4]. Comparative biology studies have played an important part in understanding how different lipid A structures drive the formation of active TLR4/MD-2 complexes. Human and horse TLR4 and MD-2 are more closely related to one another than either are to the mouse receptors, yet RSLPS is an agonist at horse TLR4/MD-2 and an antagonist at both the mouse and human receptors. In this study, we have confirmed the importance of R385, but we have also identified P442 as a key player in conferring RSLPS agonist activity at TLR4.

Acyl chain number has long been recognised as an important structural determinant of whether a given lipid A behaves as an agonist or antagonist [6], [7], [8], [9]. Lipid IVa and RSLPS are both partial agonists at horse TLR4 but have different efficacies. Relative to ECLPS (100%), lipid IVa and RSLPS have 55% and 88% efficacies respectively (figure 2A) [15]. The decreased efficacy of these lipids in comparison to ECLPS is probably caused in part by the reduced acyl chain number, which alters their positioning within the MD-2 pocket and thus the effective formation of the active TLR4/MD-2 heterotetramer. The MD-2-occupying chains of RSLA and lipid IVa probably share some, but not all, of the residue contacts formed between lipid A and MD-2, leading to a reduced signal. This subtle difference in acyl chain position is likely to underlie the different dependence of lipid IVa and RSLPS on residues 80–107 of horse MD-2, and makes identification of suitable point mutation candidates difficult. Instead, a more holistic effect of this region on signalling is likely to derive from a species difference in overall charge and/or flexibility, since the major non-conserved residues in horse/human comprise E86/N86 and M89/K89 respectively. The effect of subtle positional differences within MD-2 is also observed in signalling by lipid IVa at mouse TLR4/MD-2, for which a very small reduction in efficacy can be explained by subtle differences in ligand-receptor interaction [5].

In addition to the extensive lipid A acyl chain interactions formed with MD-2 and TLR4*, the two diglucosamine backbone phosphate groups are supportive of ligand positioning and thought to be important for agonist activity [4]. K341, K362 and K388* of human TLR4 are involved with positioning of the 1-PO_4_ of lipid A, whereas G384 is not because of its lack of a side chain. In the overlapped human/lipid A and horse/RSLA structures, R385 of horse TLR4 is situated 9.4 Å away from the 1-PO_4_ of lipid A in the human-bound position (not shown). R342, K363 and K389*, however, sit much closer to this moiety, and so probably contribute to lipid A positioning by 1-PO_4_ interaction (not shown). These residues are also located close to the 1-PO_4_ of RSLA, and so, together with R385, probably position RSLA similarly to lipid A. Apart from R385, lipid IVa 1-PO_4_ appears only to make contact with K389* (at 6.1 Å). R385 is therefore essential for lipid IVa signalling, as it provides alternative 1-PO_4_ long-range electrostatic attraction. The increased distance between RSLA 1-PO_4_ and R385 (8.5 Å) relative to lipid IVa 1-PO_4_ probably explains the difference in effect observed by R385G mutation between these two ligands (not shown).

In the human TLR4/MD-2/lipid A crystal structure, R264 and K362 of TLR4 and S118 of MD-2 all interact with lipid A 4′-PO_4_ via charge/polar interactions [4]. These charges are conserved in horse TLR4 (K264, K363 and S118) and so are also probably involved in lipid A 4′-PO_4_ positioning. Comparison with lipid IVa and RSLA demonstrates different 4′-PO_4_ positions for the ligands. Lipid A 4′-PO_4_ sits centrally between K264, K363 and S118, lipid IVa 4′-PO_4_ is skewed towards K264, and RSLA 4′-PO_4_ is pushed towards S118 of MD-2 (not shown).

One of the key structural differences between RSLA and lipid IVa is the presence of the R2″ chain. In the docking model for RSLA, this unsaturated and extended chain is predicted to be greatly bent within the horse MD-2 hydrophobic pocket (figure 4A). In the human TLR4/MD-2/lipid A crystal structure, the R2″ chain of lipid A remains straight and contacts human MD-2 deep within the pocket. Since an R3″ chain is also present in lipid A, the MD-2 pocket is occupied fully. This forces the R2 chain out of the pocket to contact TLR4*. R3″ is absent in RSLA, therefore folding of the extended R2″ may produce a similar effect on MD-2 occupation and encourage R2 protrusion. In the mouse lipid IVa crystal, the R2 chain is positioned slightly away from TLR4*, but this is not as pronounced as for the horse model. Lipid IVa also sits much higher within mouse MD-2 than it is predicted to in the horse model. This difference in positioning may negate the need for full R2 protrusion, and explain why lipid IVa is able to activate mouse TLR4/MD-2 more efficiently than it can horse. RSLA is predicted to sit much lower within MD-2 than either lipid IVa conformation, yet R2 protrusion seems to compensate for this, and allows the ligand to activate the complex strongly. R2-TLR4* interaction is therefore probably important for a strong signal at TLR4 if other interactions are sub-optimal. Lipid IVa in the horse-bound conformation fails to form either optimal 1-PO_4_/4′-PO_4_ interactions or strong R2-TLR4* interaction, which probably causes the observed lower efficacy of this ligand in the horse relative to that in the mouse [15]. Lipid A, on the other hand, satisfies both sets of interactions, explaining its high efficacy relative to the other ligands. Our study highlights the subtle interplay between van der Waals contacts and ionic interactions involved in the activation process, with compensatory effects depending on the ligand's geometry.

The difference in CD14 requirement by ECLPS and RSLPS for signalling is curious. Although CD14 presents lipid A monomers to the TLR4/MD-2 complex, the complete biological role of CD14 in LPS signalling is still not fully understood [23]. The relative independence of RSLPS from CD14 may reflect a simple tendency of the ligand to form smaller aggregates. Alternatively, differences in O-antigen between ECLPS and RSLPS may underlie a differing need for facilitation by CD14. The presence of O-antigen does not change the activity of RSLPS or RSLA in the presence or absence of CD14 (data not shown). This is in contrast to other bacterial LPS species such as *Salmonella*, for which CD14 is required in both MyD88 and TRIF-dependent signalling to smooth LPS, but only TRIF-dependent signalling to rough LPS and lipid A [24]. Further investigation using LPS from a wide variety of bacterial species is required to explore this fully.

In summary, the results of this study demonstrate differences in predicted binding and efficacy between lipid A, RSLA and lipid IVa. In modelling studies, RSLA shares more similarities in binding the active TLR4/MD-2 complex with lipid A than it does lipid IVa, which presumably precipitates different efficacies for RSLPS, ECLPS and lipid IVa. We show that very subtle differences in ligand positioning can precipitate significant pharmacological effects, and that this is likely the result of a subtle interplay between ionic interactions and van der Waals forces. We also conclude that the fluid nature of the lipid tail of LPS leads to an overall effect tangibly different to the well-defined electrostatic anchoring [25]. This study highlights the importance of regions outside the ligand and dimerisation binding area for the signalling output of TLR4, which indicates a cooperative binding mechanism. This mechanism is particularly relevant in the context of single nucleotide polymorphism analysis, and will also ultimately impact development of therapeutic TLR4 ligands. The heterogeneity of naturally-derived RSLPS is particularly relevant to the development of synthetic therapeutic ligands, and this heterogeneity may even be the key to unlocking the potential for the use of these ligands as drugs.

## Supporting Information

Figure S1 **Sequence alignment of human and horse MD-2.**
(PDF)

Figure S2 **Sequence alignment of human and horse TLR4 LRR14-17.**
(PDF)
