# Supplementary material for: Identification of Key Residues That Confer Rhodobacter sphaeroides LPS Activity at Horse TLR4/MD-2
Source: PLoS One. 2014 May 30;9(5):e98776. doi: 10.1371/journal.pone.0098776 (PMC4039514; doi:10.1371/journal.pone.0098776)
Supplement: Figure S1 — Sequence alignment of human and horse MD-2. (PDF) [file pone.0098776.s001.pdf]

Figure S1: Sequence alignment of human and horse MD-2.

```
hMD-2                  1 MLPFLFFSTLFSSIFTEAQKQYWVCNSSDASISYTYCDKMQYPISINVNP      50
                           |..|:.|.|||||||.|.::..|:|||||.||.|||||.|:.|||||:.|
eqMD-2                 1 MFSFMLFFTLFSSIFPEPEELRWICNSSDMSIWYTYCDNMKSPISINLEP      50

hMD-2                 51 CIELKRSKGLLHIFYIPRRDLKQLYFNLYITVNTMNLPKRKEVICRGSDD     100
                           |||||.::|.||:.::||||:|:||||||:|:|::..|.|||||||||||
eqMD-2                51 CIELKGTRGHLHMLFVPRRDIKKLYFNLYLTMNSLEFPMRKEVICRGSDD     100

hMD-2                101 DYSFCRALKGETVNTTISFSFKGIKFSKGKYKCVVEAISGSPEEMLFCLE     150
                           ||||||||||||||||:||||:|::|.||:|.|:.||:.|:.||.||||.
eqMD-2               101 DYSFCRALKGETVNTTVSFSFRGMRFPKGRYSCIAEAVVGNTEEALFCLN     150

hMD-2                151 FVILHQPNSN    160
                           |.:||||:.|
eqMD-2               151 FTLLHQPSFN    160
```
