# Supplementary material for: Identification of Key Residues That Confer Rhodobacter sphaeroides LPS Activity at Horse TLR4/MD-2
Source: PLoS One. 2014 May 30;9(5):e98776. doi: 10.1371/journal.pone.0098776 (PMC4039514; doi:10.1371/journal.pone.0098776)
Supplement: Figure S2 — Sequence alignment of human and horse TLR4 LRR14-17. (PDF) [file pone.0098776.s002.pdf]

Figure S2: Sequence alignment of human and horse TLR4 LRR14-17.

```
hTLR4/14-17      372 LPSLEFLDLSRNGLSFKGCCSQSDFGTTSLKYLDLSFNGVITMSSNFLGL    421
                     ||||||||||||.||||.|||::|..||.||:||||||.||:|||||:||
eqTLR4/14-17     373 LPSLEFLDLSRNRLSFKSCCSEADLKTTRLKHLDLSFNDVISMSSNFMGL    422

hTLR4/14-17      422 EQLEHLDFQHSNLKQMSEFSVFLSLRNLIYLDISHTHTRVAFNGIFNGLS    481
                     |||||||||||.|||.|:|.|||||:||.|||||:|:|||.|:|||:||.
eqTLR4/14-17     423 EQLEHLDFQHSTLKQASDFPVFLSLKNLRYLDISYTNTRVVFHGIFDGLV    482

hTLR4/14-17      482 SLEV    475
                     ||:|
eqTLR4/14-17     483 SLQV    476
```
